# The consumption of Internet child pornography and violent and sex offending

**DOI:** 10.1186/1471-244X-9-43

**Published:** 2009-07-14

**Authors:** Jérôme Endrass, Frank Urbaniok, Lea C Hammermeister, Christian Benz, Thomas Elbert, Arja Laubacher, Astrid Rossegger

**Affiliations:** 1 Department of Justice, Psychiatric/Psychological Service, Canton of Zurich, Feldstrasse 42, 8004 Zurich, Switzerland; 2 Praxis Dr. Benz, Mühlebachstrasse 42, 8008 Zurich, Switzerland; 3 University of Constance, 78457 Constance, Germany

## Abstract

**Background:**

There is an ongoing debate on whether consumers of child pornography pose a risk for hands-on sex offenses. Up until now, there have been very few studies which have analyzed the association between the consumption of child pornography and the subsequent perpetration of hands-on sex offenses. The aim of this study was to examine the recidivism rates for hands-on and hands-off sex offenses in a sample of child pornography users using a 6 year follow-up design.

**Methods:**

The current study population consisted of 231 men, who were subsequently charged with consumption of illegal pornographic material after being detected by a special operation against Internet child pornography, conducted by the Swiss police in 2002. Criminal history, as well as recidivism, was assessed using the criminal records from 2008.

**Results:**

4.8% (n = 11) of the study sample had a prior conviction for a sexual and/or violent offense, 1% (n = 2) for a hands-on sex offense, involving child sexual abuse, 3.3% (n = 8) for a hands-off sex offense and one for a nonsexual violent offense. When applying a broad definition of recidivism, which included ongoing investigations, charges and convictions, 3% (n = 7) of the study sample recidivated with a violent and/or sex offense, 3.9% (n = 9) with a hands-off sex offense and 0.8% (n = 2) with a hands-on sex offense.

**Conclusion:**

Consuming child pornography alone is not a risk factor for committing hands-on sex offenses – at least not for those subjects who had never committed a hands-on sex offense. The majority of the investigated consumers had no previous convictions for hands-on sex offenses. For those offenders, the prognosis for hands-on sex offenses, as well as for recidivism with child pornography, is favorable.

## Background

The legal definition of what constitutes child pornography, as well as the interpretation and enforcement of statutory provisions relating to child pornography, vary considerably from country to country. Not surprisingly, the definitions of child pornography to be found in the relevant research literature are equally heterogeneous [1-3]. The definitions differ regarding the age boundary of consenting adult vs. child and in respect to how explicit the sexual depiction of the material must be in order to be considered "illegal pornography". Furthermore, some criminal codes penalize only the production and distribution of illegal pornography, while others criminalize the possession as well.

In April 2002, a new article was introduced into Swiss penal law stating that the possession of pornographic material depicting sexual acts with children, excrement, animals, as well as violent sexual intercourse, is illegal (Art.197 Swiss Penal Code). Accordingly, the production, import, storage, marketing, making available, and presentation of illegal pornography is a punishable offense. Before April 2002, only the trade and production of child pornography was against the law, not its possession. Swiss penal law neither specifies the age which discriminates children from adults, nor does it specifically state the exact characteristics of pornography. However, judicial practice defines children younger than 16 as juveniles, which is also the appointed age of consent [4,5].

The high accessibility of the Internet has changed the consumption of child pornography. According to Cooper, Delmonico and Berg [6], three attributes of the Internet, called the "Triple A Engine", facilitate the consumption of child pornography: Accessibility (millions of websites are accessible 24 hours a day, 7 days a week), affordability (acquiring the material does not demand substantial financial resources), and anonymity (no personal contact with others is needed to consume child pornography). Quayle, Vaughan, and Taylor [7] also underline the importance of the ostensible anonymity of the Internet for the consumption of Internet child pornography, as it does not require contacting a dealer and the material can easily be acquired at home. Furthermore, virtual pornographic material can be stored easily and no strenuous effort is needed to keep the illegal material hidden [8].

One of the most consistent findings when trying to characterize the "typical" user of child pornography is – not surprisingly – that there are only male consumers [1,9-16]. According to Quayle, Erooga, Wright, Taylor and Harbinson [2], it is safe to assume that female child pornography consumers are non-existent. Furthermore, there is evidence that consumers of child pornography have a relatively high educational background. According to Wolak, Finkelhor and Mitchell [16], 38% out of 1,713 convicted consumers of child pornography in the U.S. had a high-school diploma, 21% went to college at some point, 16% held a college diploma, and 4% finished their education at a doctoral level. This finding was corroborated by Riegel [13], who found that 77% of his sample of alleged pornography consumers consisted of college graduates. In the Swiss study of Frei, Erenay, Dittman and Graf [1], one out of three child pornography users reported working in an executive position or holding an academic degree. Burke, Sowerbutts, Blundell and Sherry [4], as well as O'Brien and Webster [17], found higher scores of intelligence, a better educational background, as well as a higher rate of employment for Internet child pornography consumers than for hands-on sex offenders.

When investigating the prevalence of Internet child pornography consumption, an important practical question is whether consumers of child pornography pose a risk for hands-on sex offenses. Empirical studies use different study designs and samples for answering this question. However, there are three main approaches to be found in the relevant research literature.

Firstly, there are studies that examine the role of child pornography consumption on offending in samples of hands-on sex offenders [10,15]. Following such an approach, Kingston, Fedoroff, Firestone, Curry and Bradford [10] examined convicted hands-on sex offenders and found that the consumption of illegal pornography was a relevant risk factor, namely that those offenders who had consumed illegal pornography were more likely to re-offend – irrespective of their risk-level of recidivism. Howitt [9] investigated convicted hands-on sex offenders, who reported that the source of sexual stimuli did not stem solely from child pornographic material, but also from the cognitive manipulation of legal adult pornography or from seeing arousing images in newspapers and magazines (usually not involving nudity). Unlike Kingston et al. [10], Howitt [9] concluded that it is not possible to establish an association between hands-on sex offenses and the consumption of child pornographic material. Since Kingston et al. [10] and Howitt [9] investigated samples containing only hands-on offenders, it is evident, that the conclusions of these authors cannot be generalized to hands-off offenders.

Secondly, there are studies that examine the prevalence of prior convictions for hands-on sex offenses in populations of child pornography consumers [1,14,15].

Webb, Craissati and Keen [15] compared 90 child pornography consumers with 120 offenders convicted of hands-on sex offenses. The number of offenders with prior convictions for sexual offenses was higher in the group of hands-on sex offenders than in the group of child pornography users. In an investigation by Frei, Erenay, Dittmann and Graf [1], none of the child pornography consumers had a criminal record.

This approach allows an initial conclusion to be drawn regarding the prevalence of hands-on sex offenses for child pornography consumers. However, due to the limitations of retrospective designs, it cannot answer the question of whether child pornography consumption represents a risk factor for committing hands-on sex offenses in the future.

Thirdly, research designs following up on a sample of offenders convicted of child pornography consumption would appear to be the best approach [14]. So far, there is only one study that has analyzed the association between child pornography consumption and the subsequent perpetration of hands-on sex offenses [14]. In their sample of convicted child pornography consumers, Seto and Eke [14] found a recidivism rate of 1.3% for hands-on sex offenses and 5.3% for hands-off sex offenses in a follow-up time of two and a half years. In this study, 24% of the study sample had been convicted for a hands-on sex offense against a minor. An Internet based survey by Riegel [13] found results pointing in the same direction as Seto and Eke's: Participants identifying themselves as "Boy-Attracted Pedosexual Males (BPM)" were asked whether the consumption of child pornographic material increased the desire to commit sexual acts with minors, 84.5% of the survey sample replied „rarely" or „never". The author interpreted this finding as an indication that the consumption of child pornography alone is not a sufficient risk factor for committing a hands-on sex offense.

Altogether, the empirical literature does not put forward any evidence that the consumers of child pornography pose a considerably increased risk for perpetrating hand-on sex offenses. Instead, the current research literature supports the assumption that the consumers of child pornography form a distinct group of sex offenders. Though some consumers do commit hands-on sex offenses as well – the majority of child pornography users do not. Previous hands-on sex offenses are a relevant risk factor for future hands-on sex offenses among child pornography users, just as they are among sex offenders in general. The consumption of child pornographic material alone does not seem to predict hands-on sex offenses.

The aim of this study was to analyze the characteristics of a sample of child pornography users and the proportion of those who subsequently re-offended with hands-on and hands-off sex offenses.

## Methods

### Ethical approval

Non-invasive studies and studies that do not examine patients directly are not investigated by the Ethics Commission of the Canton of Zurich, as their ethical soundness is generally assumed. Nonetheless, in order to ensure ethical and judicial approval, the entire research project was presented to the Justice Department of the Canton of Zurich. A second examination was conducted by the Federal Office of Justice in connection with the request for the criminal records. The study was approved and supported by both authorities.

### Sample

On a website owned by the US company „Landslide Productions", illegal pornography was distributed over the Internet [2,18]. When the website was shut down by the US-Postal Service in 1999, it had approximately 75'000 customers worldwide. The user data was handed over to the judicial authorities in the respective countries, which led to well coordinated, international police operations [18]. Through a number of house searches in 2002, a Swiss police operation ("Operation Genesis") revealed over 400 persons suspected of having consumed illegal pornographic material via Landslide Productions Inc. Of these 400 people, only those suspected of child pornography consumption, were included in the study sample. This led to a sample size of N = 231.

### Data collection

The Department of Public Prosecution provided the judicial files and all necessary information for assessing the socio-demographic and offense related characteristics.

2002 was the cut-off point, with any convictions prior to this date being 'previous convictions'. The follow-up was in 2008, therefore resulting in a six year follow-up period, during which time all new offenses were deemed to be re-offending.

The information on previous convictions was collected from the criminal records.

Recidivism was assessed based on two sources of information, namely the criminal records as well as the database of the Zurich criminal justice system. By using these two sources of information, it was possible to analyze two different definitions of recidivism.

According to a strict definition, recidivism was assumed only if new convictions subsequent to the index offense (child pornography consumption) were registered in the criminal records.

A broader definition of recidivism was introduced including convictions, as well as ongoing investigations and charges, as it is a well known fact that the use of criminal records frequently leads to an underestimation of especially sex and violent recidivism. Thus, in Switzerland, convictions of criminally non-responsible offenders, which often involve sex and violent offenses, are not registered in the official criminal records. Also, the registration of sex and violent offenses in the criminal records often takes several years due to longer investigation and appeal periods.

### Statistical analysis

Descriptive statistical analyses were performed to examine the socio-demographic and offense related characteristics of the study sample. In order to investigate further, a stratified analysis was conducted: During the court proceedings, 95% (n = 217) of the sample confessed to having used child pornography, however, the evidence only led to convictions in 55% (n = 127) of the cases. Unlike current legal practice, saving illegal data in the temporary data cache of the computer was not enough for a conviction in 2002 – only if a permanent recording of the illegal material could be proven was it possible to convict the accused. Though only around half of the sample was actually convicted, there were sufficient grounds for assuming that all subjects in the study sample had consumed child pornography, seeing as they had registered with their personal credit cards and confessed to the allegations. Accordingly, the legal fees were also imposed on those offenders who were acquitted. However, this lead to two different subsamples which allowed a stratified analysis: the sample of convicted offenders and the sample of acquitted offenders. The aim of the stratified analysis was to determine whether a legal sanction led to a behavioral change regarding the consumption of illegal pornography. Significance was tested with the chi-square test.

All statistical analyses were carried out with STATA SE 10.1.

## Results

### Socio-demographic characteristics

At the time of offense, the average age of the subjects was 36 years (SD 10.0), with a range from 18 to 65. The majority of the subjects were Swiss nationals (94%, n = 217), 58% (n = 128) were single, 33% (n = 74) were married, 8% (n = 19) were divorced and 1% (n = 3) were widowed. 25% (n = 55) of the study sample had children.

Out of 226 subjects, 45% (n = 102) worked in a position that required a university level diploma, 50% (n = 112) held a job requiring formal vocational training and 5% (n = 12) were unskilled workers. Roughly one third (32%, n = 70) worked in a computer science or engineering-oriented profession, 26% (n = 56) held a blue collar job and 33% (n = 72) worked in the service industry. Nearly half of the study sample worked primarily with a computer at their place of work.

### Offense characteristics

#### Location of pornography consumption (index offense)

95% (n = 218) of the sample had Internet access from home and 30% (n = 68) at work. Even so, child pornography was consumed at work in only four cases (2%) and only one subject actually saved child pornography onto a work computer.

#### Characteristics of the illegal material

Two subjects (1%) were in possession of pornographic material they had made themselves. 19% (n = 43) of the sample were in possession of more than 5'000 files of illegal pornography. Forty percent (n = 93) of the subjects consumed only child pornography, the rest consumed other types of illegal pornography, such as pornography depicting sexual acts with animals, excrement, or involving brutality. One out of three subjects (33%, n = 77) consumed at least three types of illegal pornography.

### Criminal history

Before the police operation "Genesis" in 2002, 4.8% (n = 11) of the study sample had prior convictions for a sex and/or violent offense: Two subjects (1.0%) had prior convictions for hands-on sex offenses involving child sexual abuse, 3.5% (n = 8) subjects had prior convictions for hands-off sex offenses (possession/consumption of illegal pornography) and one subject had a prior conviction for a violent offense.

### Recidivism

Applying the *strict definition *of recidivism, 3.0% (n = 7) of all subjects re-offended with a sex and/or violent offense during the six year follow-up. In detail, 2.6% (n = 6) of the study sample recidivated with a hands-off sex offense (consumption of illegal pornography) and 0.4% (n = 1) with a violent offense (bodily harm). No one in the study sample was convicted of a hands-on sex offense.

Applying the *broader definition *of recidivism (including reconvictions, ongoing investigations and charges), the rate of violent and/or sexual recidivism was 6% (n = 14). Nine (3.9%) of the subjects were investigated, charged or convicted for hands-off sex offenses, all of which were due to illegal pornography possession. Two subjects (0.8%) were being investigated, charged or convicted for a hands-on sex offense, namely child sexual abuse. Recidivism with a violent offense was documented in 1.3% (n = 3) of the cases.

Of the 9 subjects who had recidivated with a hands-off sex offense according to the broad definition, none had previously been convicted (before the index offense) for a hands-off sex offense. Of the two subjects who had re-offended with a hands-on sex offense according to the broad definition, one had previously been convicted for a hands-on sex offense.

Table 1 shows the prevalence rates for prior convictions, as well as recidivism rates.

**Table 1:** Criminal history and recidivism divided into offense categories

	Criminal history (N = 231)	Recidivism (N = 231)	
		
		Strict definition	Broad definition
		
	% (n)	% (n)	% (n)
Hands-on sex offenses	1.0 (2)	0 (0)	0.8 (2)
Hands-off sex offenses	3.5 (8)	2.6 (6)	3.9 (9)
Violent offenses	0.4 (1)	0.4 (1)	1.3 (3)

**Sex and/or violent offenses**	**4.8 (11)**	**3.0 (7)**	**6.0 (14)**

#### Convicted vs. acquitted consumers of illegal pornography

Stratified analyses of the data revealed several differences between subjects convicted of consuming illegal pornography (group: convicted) and those who were acquitted (group: acquitted).

Acquitted offenders were significantly more likely to be married (40% vs. 28%) but did not differ with respect to vocational education or job position.

More frequently, various forms of illegal pornography were found in the group of convicted consumers and these subjects were also more likely to own a collection of this material (30% vs. 5%). Furthermore, they held more subscriptions to commercial websites containing legal pornographic material (28% vs. 10%) and were more likely to be in the possession of commercial (legal) pornographic material recorded on VHS, CDs, and DVDs (19% vs. 4%).

Convicted and acquitted subjects did not differ with respect to prior convictions. However, the two subjects already convicted for a hands-on sex offense, along with the only person previously convicted for a violent offense, were not convicted for the possession of illegal pornography as result of the police operation.

According to the broad definition of recidivism, 7.3% (n = 9) of the convicted group re-offended with a hands-off sex offense while none of the acquitted group did. Vice-versa, none of the convicted group re-offended with a hands-on sex offense while 1.9% (n = 2) of the acquitted group did.

Table 2 shows the significant differences in the consumption pattern of convicted vs. acquitted users of illegal pornography.

**Table 2:** Significant differences in the consumption pattern of convicted vs. acquitted users of illegal pornography

		Convicted	Acquitted	χ^2^
		
		% (n)	% (n)	
Type of consumed illegal pornography	Child	86 (109)	48 (50)	37.98*
	Brutality	47 (60)	25 (26)	12.11*
	Excrement	45 (57)	32 (33)	4.16*
	Animals	44 (56)	28 (29)	6.46*
Collection of illegal pornography		30 (38)	5 (5)	23.8*
Subscription to pornographic websites		100 (127)	94 (96)	7.67*
Possession of commercial VHS tapes		28 (36)	10 (10)	12.3*
Possession of commercial CDs and DVDs		19 (24)	4 (4)	12.14*

*p < .05

## Discussion

For the present study, the criminal records of all men charged with child pornography, as a result of the police operation "Genesis", were analyzed. This study is one of the few scientific reports covering a six year follow-up period of a sample of subjects charged with the consumption of child pornographic material.

Descriptive analyses suggest that child pornography users are less likely to be married: While only one out of three subjects was married at the time of the police operations, the census data of the Canton of Zurich reported that 45% of the Swiss male population living in the Canton of Zurich was married. Moreover, the foreign nationals (6%) in the present study were underrepresented in comparison to the 31% of foreign male residents in the Canton of Zurich [19]. This finding is remarkable seeing as this underrepresentation cannot be found for hands-on sex offenses [20]. As in other studies [4,13,16,17], the finding that child pornography consumers are well educated was replicated in the present study: Only 5% of the investigated sample held an unqualified job position – a figure that is rather low compared to the 16% of unskilled workers in the permanent living population of the Canton of Zurich [21].

In summary, our results suggest that users of child pornography are probably well integrated in Swiss society, as the majority of subjects held a job requiring extensive training and were Swiss nationals.

The question of whether consumers of child pornography pose a risk for hands-on sex offenses has not yet been answered. In their sample of child pornography users, Seto und Eke [14] found that 24% of the study sample had a criminal record for a hands-on sex offense against a minor and 15% had a prior conviction for the possession of child pornography. Other studies however, have found low rates of previous convictions for hands-on sex offenses among child pornography consumers [4,15].

In our study, we were able to replicate the finding that the majority of child pornography consumers do not have a criminal record for a violent and/or sex offense. Before the police operation, 3.5% of the study sample had prior convictions for a hands-off sex offense and 1% (n = 2) for hands-on sex offenses involving child sexual abuse. We cannot explain the large difference of prior conviction rates in our sample compared to Seto and Eke [14] – further research would be necessary to find out if they differ significantly in regard to socio-economic, educational and/or other variables.

Studies investigating hands-on sex offender populations support the assumption that the consumption of illegal pornography is a relevant risk factor for recidivism [10]. In contrast, Seto and Eke [14] found low recidivism rates for both hands-on (1.3%) and hands-off (5.3%) sex offenses in a follow-up time of two and a half years for their sample of child pornography consumers.

Similar to Seto and Eke [14], we found low rates of recidivism among our sample. When applying the broader definition of recidivism by taking investigations and charges into account, the recidivism rates were 0.8% for hands-on and 3.9% for hands-off sex offenses. These recidivism rates after a follow-up time of six years indicate that the risk of re-offending for child pornography consumers is quite low.

### Limitations

The investigated population could be severely biased, seeing as the users needed a credit card and sufficient knowledge of a foreign language (English) in order to gain access to the pornographic material. This could explain the rather elevated proportion of well educated subjects in the study sample and indicates that the investigated sample is most probably not representative for consumers of illegal pornography in Switzerland.

## Conclusion

Among the subjects of the present study, only 1% were known to have committed a past hands-on sex offense, and only 1% were charged with a subsequent hands-on sex offense in the 6 year follow-up. The consumption of child pornography alone does not seem to represent a risk factor for committing hands-on sex offenses in the present sample – at least not in those subjects without prior convictions for hands-on sex offenses.

## Competing interests

The authors declare that they have no competing interests.

## Authors' contributions

JE has given substantial contributions to the conception, analysis, and interpretation of the data and helped draft the manuscript. FU and CB developed the idea for the study and developed the study design. LH was involved in the process of data collection and performed the statistical analysis. TE and AL have been involved in revising the manuscript critically. AR was substantially involved in interpreting the results and writing the manuscript. All authors read and approved the final manuscript.

## Pre-publication history

The pre-publication history for this paper can be accessed here:



## References

[B1] Frei A, Erenay N, Dittmann V, Graf M (2005). Paedophilia on the Internet – a study of 33 convicted offenders in the Canton of Lucerne. Swiss Medical Weekly.

[B2] Quayle E, Erooga M, Wright L, Taylor M, Harbinson D, Quayle E, Erooga M, Wright L, Taylor M, Harbinson D (2006). Abuse Images and the Internet. Only Pictures? Therapeutic work with Internet sex offenders.

[B3] Taylor M, Quayle E, Holland G (2001). Child Pornography, the Internet and offending. The Canadian Journal of Policy Research.

[B4] Burke A, Sowerbutts S, Blundell B, Sherry M (2002). Child Pornography and the Internet: Policing and Treatment Issues. Psychiatry, Psychology and Law.

[B5] Kuhnen K (2007). Kinderpornographie und Internet; Child pornography in the Internet.

[B6] Cooper A, Delmonico DL, Burg R (2000). Cybersex users, abusers, and compulsives: New findings and implications. Cybersex: The dark side of the force A special issue of the Journal of Sexual Addiction & Compulsivity.

[B7] Quayle E, Vaughan M, Taylor M (2006). Sex offenders, internet child abuse images and emotional avoidance: The importance of values. Aggression and Violent Behavior.

[B8] Quayle E, Erooga M, Wright L, Taylor M, Harbinson D, Quayle E, Erooga M, Wright L, Taylor M, Harbinson D (2006). Collecting Images. Only Pictures? Therapeutic work with Internet sex offenders.

[B9] Howitt D (1995). Pornography and the paedophile: Is it criminogenic?. Br J Med Psychol.

[B10] Kingston DA, Fedoroff P, Firestone P, Curry S, Bradford JM (2008). Pornography use and sexual aggression: the impact of frequency and type of pornography use on recidivism among sexual offenders. Aggressive Behavior.

[B11] Quayle E, Taylor M (2001). Child seduction and self-representation on the Internet. Cyberpsychol Behav.

[B12] Quayle E, Taylor M (2002). Child pornography and the Internet: Perpetuating a cycle of abuse. Deviant Behavior.

[B13] Riegel DL (2004). Effects on boy-attracted pedosexual males of viewing boy erotica. Arch Sex Behav.

[B14] Seto MC, Eke AW (2005). The criminal histories and later offending of child pornography offenders. Sexual abuse: a journal of research and treatment.

[B15] Webb L, Craissati J, Keen S (2007). Characteristics of internet child pornography offenders: a comparison with child molesters. Sexual abuse: a journal of research and treatment.

[B16] Wolak J, Finkelhor D, Mitchell KJ (2005). Child-Pornography Possessors Arrested in Internet-Related Crimes: Findings From the National Juvenile Online Victimization Study.

[B17] O'Brien MD, Webster SD (2007). The construction and preliminary validation of the Internet Behaviours and Attitudes Questionnaire (IBAQ). Sex Abuse.

[B18] Quayle E, Calder MC (2004). The Impact of Viewing on Offending Behaviour. Child Sexual Abuse And the Internet: Tackling the New Frontier.

[B19] Statistisches Amt des Kantons Zürich (2008). Wohnbevölkerung nach Heimat und Geschlecht. http://www.statistik.zh.ch/themenportal/themen/daten_detail.php?id=572&tb=1&mt=2.

[B20] Endrass J, Rossegger A, Urbaniok F (2007). Zürcher Forensik Studie. Abschlussbericht zum Modellversuch „Therapieevaluation undPrädiktorenforschung". http://www.zurichforensic.org.

[B21] Statistisches Amt des Kantons Zürich (2008). Erwerbsbevölkerung nach Ausbildung, 2007. http://www.statistik.zh.ch/themenportal/themen/daten_detail.php?id=564&tb=2&mt=2.

